# Dysregulated Sialylation in Cancer: From Immunosuppressive Microenvironment to Siglec-Targeted Therapeutics

**DOI:** 10.3390/biom15101375

**Published:** 2025-09-27

**Authors:** Yuecheng Zhang, Zhengyao Gao, Yuhan Zhang, Siqin Ai, Wenyan Li, Lingbo Sun

**Affiliations:** 1 Key Laboratory of Analytical Technology and Detection of Yan’an, College of Chemistry and Chemical Engineering, Yan’an University, Yan’an 716000, China; yuechengzhang@yau.edu.cn; 2 Medical College of Yan’an University, Yan’an University, Yan’an 716000, China; zhenyaogao@stu.yau.edu.cn (Z.G.); yuhanzhang@peihua.edu.cn (Y.Z.); 3 Department of Medical Laboratory Technology, School of Medical Technology, Xi’an Medical College, Xi’an 710309, China; aisiqin@xagdyz.com; 4 Medical College of Xi’an Pei Hua University, Xi’an Pei Hua University, Xi’an 710125, China

**Keywords:** sialic acid, sialic acid-siglec axis, TME, therapeutic

## Abstract

Sialic acid, typically positioned at the terminal ends of glycoprotein or glycolipid chains via glycosyltransferase activity, is indispensable for intercellular recognition and signal transduction. Aberrant sialylation has been implicated in disrupted cell communication and oncogenic signaling, contributing to carcinogenesis. Consequently, targeting sialic acid metabolism has emerged as a promising strategy for cancer diagnosis and therapy. This review first delineates the physiological biosynthesis of sialic acid and molecular mechanisms underlying its pathological dysregulation. We then examine the sialic acid–Siglec axis as an immune checkpoint in cancer immunotherapy, highlighting its functional convergence and divergence from the PD-1/PD-L1 pathway. Furthermore, we elucidate how aberrant sialylation drives malignant transformation. Finally, we synthesize current therapeutic strategies targeting the sialic acid–Siglec axis, with particular emphasis on implementing nanomaterial-based platforms in clinical translation. These advances may yield novel diagnostic tools and therapeutic targets for glycobiology-guided precision medicine.

## 1. Introduction

Cancer is a disease characterized by aberrant cellular proliferation, with its tumorigenesis and progression representing a multistep, complex process involving diverse molecular and cellular events. Post-translational modifications (PTMs)—covalent alterations of amino acid side chains in translated proteins—play a crucial role in regulating protein activity, localization, stability, and interaction networks [1]. Common PTMs include phosphorylation, acetylation, methylation, ubiquitination, SUMOylation, glycosylation, etc.

Glycosylation is a ubiquitous and highly diverse PTM in eukaryotic cells, characterized by the covalent attachment of glycans to proteins or lipids [2,3]. This process occurs primarily in the endoplasmic reticulum (ER) and Golgi apparatus, where glycosyltransferases and glycosidases sequentially assemble, modify, and trim carbohydrate structures. Mammalian glycans incorporate the following key monosaccharides: glucose (Glc), galactose (Gal), N-acetylglucosamine (GlcNAc), N-acetylgalactosamine (GalNAc), mannose (Man), fucose (Fuc), xylose (Xyl), glucuronic acid (GlcA), iduronic acid (IdoA) and sialic acid (predominantly N-acetylneuraminic acid, Neu5Ac) [4].

Glycosylation is classified into distinct types, which mainly include N-linked glycosylation (Asn residues), O-linked glycosylation (Ser/Thr residues), glycolipids (glycan-linked lipids), etc. [5]. The fidelity of distinct glycosylation types is critically dependent on multiple regulatory factors including substrate availability, precise enzyme localization, stringent transcriptional control, and compartmentalized organelle-specific trafficking. Aberrant glycosylation profoundly influences critical cellular processes including proliferation, immune evasion, and metastatic potential [4]. Characteristic pathological alterations encompass: (1) disrupted N-/O-linked glycosylation pathways, (2) impaired glycolipid/glycoprotein metabolism, and (3) dysregulated sialylation—particularly the tumor-associated overexpression of sialyl Lewis antigens (SLe^a^, SLe^x^) [6]. As the terminal residue of most vertebrate glycans, sialic acid (catalyzed by glycosyltransferases) mediates cell–cell recognition and signaling [7,8]. Malignant progression is marked by two hallmark sialylation-related phenomena: First, hypersialylation drives immune escape through multiple mechanisms—either by suppressing NK cell and macrophage effector functions [9,10,11] or by concealing tumor-associated antigens (e.g., STn expression in early carcinogenesis) [12,13]. Second, pathological sialylation contributes to systemic dysfunction including: (i) compromised serum glycoprotein activity (affecting coagulation and immune responses) [14], (ii) neurodevelopmental impairments via altered neurotransmitter receptor glycosylation [15], and (iii) sustained inflammatory and autoimmune conditions [16]. These disease-associated modifications establish sialylation signatures as clinically valuable biomarkers for malignancies, infectious processes, and inflammatory disorders.

In summary, the importance of abnormal sialylation is reflected in its profound effects on cell recognition, signaling, protein function, molecular transport, immune regulation, and pathophysiological processes. Understanding and studying abnormal sialylation can not only help to reveal the mechanism of the disease but also explore new targets for diagnosis and treatment. Targeting sialic acid for cancer diagnosis and treatment is a promising research direction. Although sialylation has been shown to be a therapeutic target for cancer treatment, its associated immunosuppression often results in poor treatment outcomes. Therefore, we start from the synthesis pathway of sialic acid again, aiming to understand the normal synthesis pathway of sialic acid in cells and the specific mechanism of abnormal changes. This article describes the significance of sialic acid–Siglec as a checkpoint for cancer immunotherapy. Next, we combined with recent advances to provide a more comprehensive picture of how changes in sialylation lead to the development of cancer cells. Finally, we summarize the therapeutic approaches targeting sialic acid and specifically describe the role of novel nanomaterials in the clinical treatment of sialic acid. We aim to provide new diagnostic options or therapeutic targets for glycan-mediated therapeutic interventions.

## 2. The Normal Synthetic Pathway of Sialic Acid

**Figure 1:**
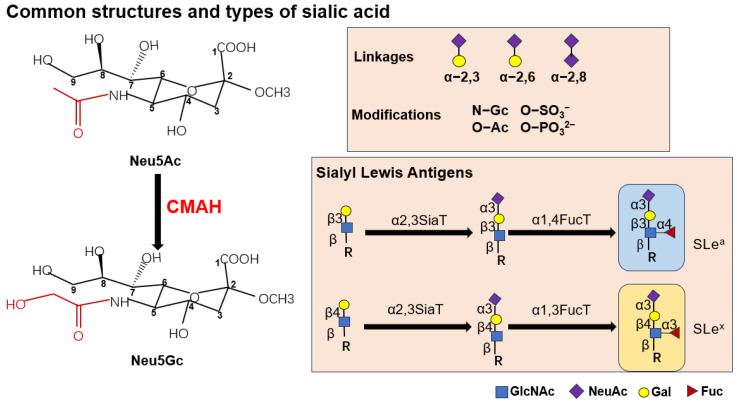
Common structures and types of sialic acid. Neu5Ac and Neu5Gc are similarly structured, and they possess different groups at the C-5 position, which are expressed in red. Neu5Ac is converted to Neu5Gc with the participation of CMAH enzyme. The common linkage of sialic acid includes α-2,3-, α-2,6- and α-2,8-linked. SLe^a^ and SLe^x^ are common forms of sialic acid associated with tumors, which are synthesized in response to different substrate-specific sialyltransferases.

**Figure 2:**
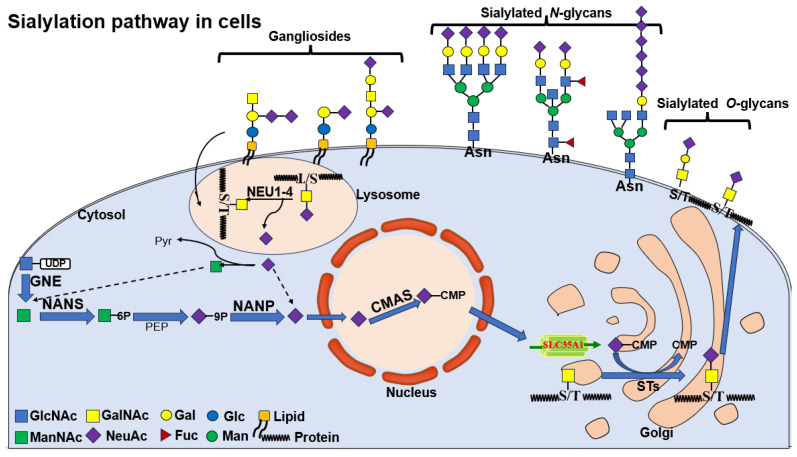
Sialylation pathway in cells. The nucleotide glycan UDP-GlcNAc, the product of hexosamine pathway, is converted into ManNAc by UDP-GlcNAc 2-epimerase (whose encoding gene is GNE in humans). ManNAc is metabolic precursor for the synthesis of sialic acid and produces Neu5Ac in the cytosol, which then enters the nucleus to produce CMP-Neu5Ac. CMP-Neu5Acs are transported into Golgi where they are used by sialyltransferase to produce glycoproteins or glycolipids, respectively. Finally, sialic acids are recycled by neuraminidases, regenerating sialic acid monomers that can be re-used. The figure shows three types of sialylated glycans, which involve sialylated N-glycans, sialylated O-glycans and gangliosides.

Sialic acids constitute a family of nine-carbon α-keto acid sugars (C1–C9) derived from neuraminic acid, with structural diversity arising from natural modifications at specific carbon positions, predominantly including N-acetylneuraminic acid (Neu5Ac), N-glycolylneuraminic acid (Neu5Gc), and 2-keto-3-deoxynonulosonic acid (KDN) in mammalian systems [17] (Figure 1). These negatively charged sugars typically occupy terminal positions on glycoconjugates, where their biosynthesis initiates through a four-step cytoplasmic pathway: (1) UDP-GlcNAc is converted to ManNAc-6-P by the bifunctional GNE enzyme (mutations in which cause sialuria and inclusion body myopathy); (2) Neu5Ac-9-P is synthesized by NANS through condensation of ManNAc-6-P with phosphoenolpyruvate; (3) NANP-mediated dephosphorylation yields free Neu5Ac; and (4) CMAS catalyzes CMP-Neu5Ac formation in the nucleus for subsequent Golgi transport via SLC35A1 [18,19] (Figure 2). Humans uniquely lack functional CMAH, restricting endogenous production to Neu5Ac while enabling dietary incorporation of Neu5Gc from red meat and dairy sources [20,21] with pathological consequences including oncogenic effects evidenced by elevated Neu5Gc in malignancies correlating with dietary intake and promotion of hepatocellular carcinogenesis in CMAH-deficient models [22]. The Golgi apparatus facilitates diverse sialic acid modifications (O-acetylation, O-methylation, O-sulfation) and linkage variations (α2-3, α2-6, α2-8, or alternating α2-8/α2-6) through over 20 sialyltransferase subtypes, including polysialic acid (PolySia) formation via α2-8 linkages [18]. Degradation occurs through four spatially distinct neuraminidases (NEU1-lysosomal, NEU2-cytosolic, NEU3-plasma membrane, NEU4-mitochondrial) that cleave sialic acids into ManNAc and pyruvate for recycling or excretion, with dysregulation contributing to pathological states ranging from carcinogenesis (through immune evasion mediated by hypersialylation) to infectious susceptibility (via viral exploitation of sialic acid variants for cellular entry) [23]. These complex biosynthetic, modificative, and catabolic pathways underscore the critical role of sialic acid biology in health and disease.

## 3. Abnormal Sialic Acid Synthesis

Abnormal sialic acid synthesis, characterized by (1) enhanced glycan substrate availability, (2) upregulated sialyltransferase expression, and (3) diminished sialidase activity, serves as a hallmark of cellular malignant transformation. This triad of abnormalities drives premature termination of glycosylation pathways and consequent hypersialylation, manifesting as elevated sialylated glycans on tumor cell surfaces. The dynamic balance of sialylation is precisely regulated by two counteracting enzyme systems, which are sialyltransferases that catalyze sialic acid addition and sialidases (neuraminidases) that mediate its removal from glycoconjugates. The stoichiometry of these opposing enzymatic activities ultimately determines cellular sialic acid content, with tumorigenesis frequently exhibiting a characteristic shift toward net sialylation through both increased synthetic capacity (sialyltransferase overexpression) and impaired degradation (sialidase suppression).

### 3.1. The Impact of Sialyltransferases in Cancer Progression and Therapy

Different sialyltransferase families are associated with distinct cancer types (Table 1). Altered sialyltransferase activity significantly influences cancer cell behavior through multiple mechanisms. A key example is ST6GalNAcI, whose upregulation drives synthesis of the STn antigen by transferring α2,6-linked sialic acid to O-GalNAc residues [24]. This enzyme exhibits unique biological significance as the sole sialyltransferase capable of STn biosynthesis in human cells. Elevated ST6GalNAcI expression in breast and prostate cancer models directly induces STn expression and promotes metastatic progression through epithelial–mesenchymal transition (EMT) activation [25,26]. The oncogenic effects of ST6GalNAcI extend across multiple cancer types through diverse molecular mechanisms. For example, it activates STAT5b to upregulate IGF-1 expression in gastric cancer, suggesting therapeutic potential for metastatic disease [27]. In ovarian cancer stem cells, its silencing attenuates malignant properties including proliferation, migration, invasion, and tumorigenicity [28]. When combined with atezolizumab, ST6GalNAcI may serve as a highly sensitive diagnostic biomarker for lung cancer detection [29].

The ST3Gal sialyltransferase family similarly contributes to cancer progression in ovarian cancer [30,31]. Another critical enzyme, ST6GAL1, demonstrates widespread oncogenic activity through its overexpression in multiple malignancies including prostate cancer, pancreatic ductal adenocarcinoma, osteosarcoma, hepatocellular carcinoma, and glioblastoma [32,33,34,35,36]. ST6GAL1 additionally promotes therapy resistance by suppressing apoptosis in colorectal carcinoma stem cells, rectal cancer, and pancreatic cancer models [37,38,39], highlighting its potential as a therapeutic target across diverse cancer types.

**Table 1:** Human Sialyltransferases.

Sialyltransferase Family	Sialyltransferase	Preferred Acceptor Saccharide	Glycan Specificity	Type of Cancer	Regulation	References
ST3Gal	ST3Gal-I	Galβ1,3GalNAc	O-glycan	BLCA, LIHC, BRCA	Uplation	[40,41,42]
	ST3Gal-II	Galβ1,3GalNAc	O-glycan	OV	Uplation	[43]
	ST3Gal-III	Galβ1,3(4)GlcNAc	O-glycan, N-glycan	PAAD	Uplation	[44]
	ST3Gal-IV	Galβ1,4(3)GlcNAc	N-glycan, O-glycan	PAAD	Uplation	[44]
	ST3Gal-V	Galβ1,4Glc-ceramide	Glycolipid	BLCA	Downlation	[45]
	ST3Gal-VI	Galβ1,4GlcNAc	N-glycan, Glycolipid	COAD, BLCA, STAD, LUAD, MMLIHC	Downlation	[46,47,48,49,50]
					Uplation	[51]
ST6Gal	ST6Gal-I	Galβ1,4GlcNAc	N-glycan	COAD, READ, OV, PRAD, LIHC, NSCLC	Uplation	[38,52,53,54,55]
	ST6Gal-II	Galβ1,4GlcNAc	N-glycan	THCA	Uplation	[56]
ST6GalNAc	ST6GalNAc-I	GalβNAcα1,O-Ser/Thr Galβ1,3GalNAcα1, O-Ser/Thr	O-glycan	LIHC, OV, COAD, BRCA, PRAD, ESCA	Uplation	[25,28,57,58,59]
					Downlation	[60]
	ST6GalNAc-II	Galβ1,3GalNAcα1, O-Ser/Thr	O-glycans	COAD, READ, BRCA	Uplation	[61]
					Downlation	[62]
	ST6GalNAc-III	Siaα2,3Galβ1,3GalNAc	O-glycans	NSCLC	Downlation	[54]
	ST6GalNAc-IV	Siaα2,3Galβ1,3GalNAc	O-glycans	THCA, LIHC	Uplation	[63,64]
	ST6GalNAc-V	GM1b	Glycolipid	PRAD	Downlation	[65]
	ST6GalNAc-VI	All α-series gangliosides	Glycolipid	BLCA	Downlation	[66]
ST8Sia	ST8Sia-I	Siaα2,3Galβ1,4Glc-ceramide	Glycolipid	BRCA	Uplation	[67]
	ST8Sia-II	Siaα2,3Galβ1,4GlcNAc	N-glycan on NCAMa	SCLC	Uplation	[68]
	ST8Sia-III	Siaα2,3Galβ1,4GlcNAc	N-glycan on NCAMa	---	---	---
	ST8Sia-IV	(Siaα2,8)nSiaα2,3Galβ1-R	N-glycan on NCAM	---	---	---
	ST8Sia-V	GM1b, GT1b, GD1a, GD3	Glycolipid	---	---	---
	ST8Sia-VI	Siaα2,3(6)Gal	Sialic acid on O-glycan	NBL	Uplation	[69]

### 3.2. Abnormal Sialidase Activity in Disease

The four human sialidases (NEU1–NEU4) exhibit distinct substrate specificities, leading to diverse roles in pathological conditions. Among them, NEU1 is implicated in multiple diseases, including lysosomal storage disorders, infections, cancers, and neurodegenerative disorders, due to its regulation of critical signaling pathways, making it a potential therapeutic target for cancer and immune-related diseases [70]. In ovarian cancer, NEU1 is highly expressed, and its knockdown via siRNA suppresses tumor cell proliferation, invasion, and apoptosis by disrupting lysosomal and oxidative phosphorylation pathways [71]. Additionally, NEU1 has emerged as a potential target in melanoma, autism spectrum disorders, and respiratory diseases [72,73,74]. NEU2 modulates cancer stemness, as demonstrated by its desialylation-mediated inhibition of Sonic Hedgehog signaling, reducing stem-like properties in pancreatic cancer spheroid cells [75]. NEU3 overexpression enhances tumor aggressiveness in bladder cancer [76] and confers resistance to apoptosis in colon cancer [77]. Conversely, NEU4 is downregulated in hepatocellular carcinoma, where its tumor-suppressive function involves CD44 desialylation to inhibit cell migration [78]. These findings highlight the critical and varied roles of sialidases in disease progression, positioning them as promising targets for therapeutic intervention.

## 4. Significance of Targeting the Sialic Acid–Siglec Axis

The sialic acid-binding lectin family comprises three major groups: selectins (P-, L-, and E-selectin), Factor H, and sialic acid-binding immunoglobulin-like lectins (Siglecs). Selectins, belonging to the C-type lectin family, mediate cellular interactions through recognition of SLe^x^ structures, playing critical roles in leukemia cell trafficking and cancer metastasis. Factor H serves as a key regulatory protein in the alternative complement pathway, modulating immune responses. The Siglec family, consisting of over 14 type I transmembrane lectins expressed on nearly all immune cell types, specifically recognizes diverse sialoglycan structures and regulates immune cell signaling [79]. These sialic acid-binding proteins collectively form the “Sialic–Siglec axis,” which has emerged as a promising therapeutic target due to its involvement in immune modulation, cancer progression, and inflammatory processes. The differential expression patterns and glycan-binding specificities of these lectins offer opportunities for developing selective interventions in malignancies, autoimmune disorders, and infectious diseases.

### 4.1. Siglec Family Classification and Function

The Siglec family comprises sialic acid-binding immunoglobulin-like lectins that serve as transmembrane receptors primarily expressed on immune cells, mediating cell–cell interactions through recognition of sialylated glycoproteins and glycolipids. These receptors play crucial roles in immune regulation by modulating adhesion, cell signaling, and endocytosis processes [80,81,82]. The human Siglec family includes 14 members divided into two evolutionary groups: (1) conserved Siglecs (Siglec-1/CD169, -2/CD22, -4/MAG, and -15) sharing 25–30% sequence identity and (2) rapidly evolving CD33-related Siglecs (Siglec-3/-5/-6/-7/-8/-9/-10/-11/-14/-16) with 50–99% sequence identity that have diversified through gene duplication and exon shuffling [83,84].

**Figure 3:**
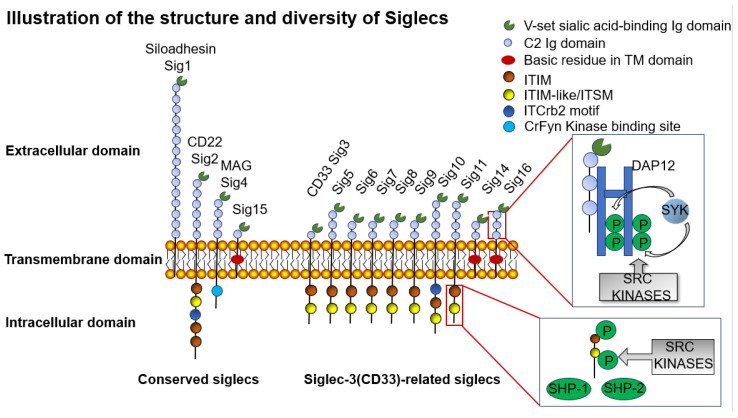
Illustration of the structure and diversity of Siglecs. There are two main groups of Siglecs: those that are highly conserved, as shown on the left, and a more diverse group of CD33-related Siglecs, as shown on the right. All Siglecs have an extracellular V-type Ig domain and at least one C2-type Ig domain. Many Siglecs also contain at least one cytoplasmic ITIM domain, involved in immunosuppressive signaling. The 3 Siglecs (Siglecs14/15/16) have a positive charge in their transmembrane domains, mediating association with DAP12 to activate immune responses. This causes recruitment and activation of SYK, leading to phosphorylation of protein kinases of downstream targets resulting in cell activation, while the remaining 9 Siglecs carry ITIM motifs, whose domains are phosphorylated by Src family kinases, which subsequently lead to the recruitment of SH2 domains containing phosphatases SHP-1 and/or SHP-2, which dephosphorylate downstream components of the immunostimulatory pathway, thereby inhibiting the immune response.

Structurally, Siglecs feature an N-terminal V-set immunoglobulin domain for sialic acid binding, variable C2-set Ig domains, and intracellular signaling motifs [85]. Functionally, they can be categorized as the following 3 types: (1) inhibitory Siglecs (9 members) containing immunoreceptor tyrosine-based inhibitory motifs (ITIMs) that recruit SHP-1/SHP-2 phosphatases to suppress immune responses; (2) activating Siglecs (Siglec-14/15/16) with transmembrane charges that associate with DAP10/12 adaptors to trigger SYK-mediated activation through MAPK and AKT pathways; (3) non-signaling Siglecs (Siglec-1/-4) lacking intracellular domains (Figure 3).

Siglec–sialoglycan interactions occur in both *cis* (same cell) and *trans* (adjacent cell) configurations, with *cis*-binding predominating due to high local sialic acid concentrations on immune cells [86]. These interactions establish self-recognition through sialic acid-based molecular patterns (SAMPs) that maintain immune homeostasis [87], contrasting with pathogen-triggered immune activation. Dysregulation of this system contributes to pathological conditions, including cancer, which develop through tumor cell hypersialylation, autoimmune disorders such as IgA nephropathy, and inflammatory diseases such as inflammatory bowel disease [88]. The dual roles of Siglecs in immune regulation and disease pathogenesis highlight their potential as therapeutic targets for immune modulation and cancer treatment.

### 4.2. Generation of Immunosuppressive Microenvironment

The tumor microenvironment (TME) represents a complex ecosystem where immune cell dysfunction promotes malignant tumor progression, invasion, and metastasis [89]. Siglecs, expressed on various immune cell subsets, serve as important phenotypic markers whose dysregulated expression in TME contributes to tumor development [90,91] (Table 2). These receptors facilitate tumor immune escape through interactions with tumor-associated sialoglycans, creating broad immunosuppressive effects across multiple immune cell populations.

Myeloid cells, the predominant immune population in solid tumors, play central roles in establishing immunosuppressive TME [103] and exhibit remarkable plasticity—macrophages, dendritic cells (DCs), monocytes and granulocytes dynamically adapt to TME signals, promoting tumor proliferation, angiogenesis and immune suppression [104,105]. Sialic acid has emerged as a critical regulator of myeloid cell polarization toward pro-tumor phenotypes, making it an attractive target for cancer immunotherapy [10,92].

**Figure 4:**
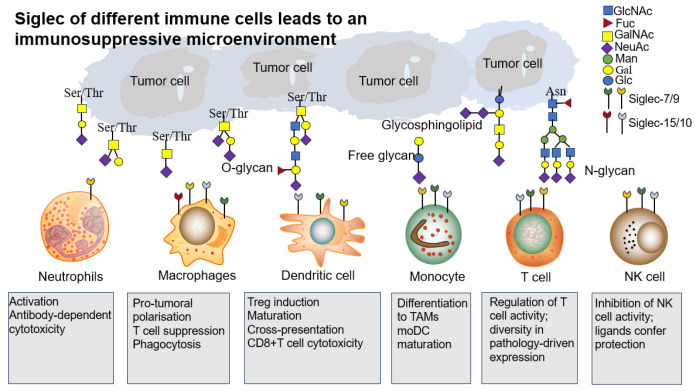
Siglec of different immune cells leads to an immunosuppressive microenvironment. Schematic of the Siglec–sialic acid axis shaping myeloid cell and lymphocyte cell function. Siglec-7/9/10/15 expression and their sialic acid ligand in the TME.

The Siglec–sialic acid axis serves as a fundamental regulatory mechanism in TME, with Siglec-7/-9/-10/-15 showing particularly strong expression on tumor-associated myeloid cells, NK cells and T cell subsets [93,95,106,107]. This molecular interaction profoundly influences immune function by modulating monocyte differentiation, macrophage polarization, DC activation, neutrophil effector functions, and NK/T cell anti-tumor activity. Current evidence demonstrates Siglec-mediated immunosuppression occurs through multiple mechanisms: myeloid cell reprogramming via sialic acid–Siglec interactions promotes alternative macrophage activation and DC dysfunction; lymphocyte inhibition through Siglec engagement dampens NK and T cell cytotoxic responses; and certain Siglecs function as novel immune checkpoints in TME. These findings highlight the therapeutic potential of disrupting the sialic acid–Siglec axis to reverse immunosuppression and restore anti-tumor immunity, offering new opportunities for cancer immunotherapy development. The ability of Siglecs to regulate multiple immune cell populations within TME positions them as promising targets for combination therapies aiming to overcome current limitations in cancer treatment (Figure 4).

#### 4.2.1. Tumor-Associated Macrophages (TAMs)

TAMs primarily originate from peripheral blood monocytes that undergo M2-polarized differentiation under the influence of tumor-derived signals and microenvironmental cues. During this process, macrophages develop tissue-specific Siglec expression patterns. In pancreatic ductal adenocarcinoma, upregulated ST3GAL1 and ST3GAL4 sialyltransferases promote monocyte-to-macrophage differentiation through Siglec-mediated signaling pathways [95]. Siglec-9 plays a particularly critical role in this differentiation process, and its engagement further suppresses anti-tumor T cell responses, contributing to immune evasion [108]. Additionally, Muc1-associated sialoglycan truncation drives macrophages toward a TAM-like phenotype characterized by elevated programmed death protein 1 (PD-L1) expression, effectively creating an immunosuppressive checkpoint [93]. These mechanisms collectively demonstrate how sialic acid–Siglec interactions shape TAM polarization and function within the tumor microenvironment, ultimately facilitating immune escape and tumor progression. The dual role of specific Siglecs in both macrophage differentiation and subsequent T cell suppression highlights their potential as therapeutic targets for reprogramming the immunosuppressive TME.

#### 4.2.2. Neutrophils

Neutrophils, while essential for innate immunity and pathogen clearance, adopt immunosuppressive functions in cancer and chronic inflammation through sialic acid–Siglec interactions. Tumor-associated neutrophils express multiple Siglecs (Siglec-5, -7, -9, and -10) that engage with hypersialylated tumor cells, leading to functional inhibition [109]. The Sialic–Siglec-9 axis in particular suppresses antibody-dependent cellular cytotoxicity against tumors, representing a key immune evasion mechanism. Recent studies reveal that Siglec-F+ splenic neutrophils induce systemic immunosuppression post-infection [110], while Siglec signaling broadly restrains neutrophil effector functions—including degranulation, chemotaxis, reactive oxygen species production, and cytokine release—through inhibitory phosphorylation cascades. Beyond intrinsic suppression, Siglec-activated neutrophils secrete TGF-β and IL-10 to dampen NK cell cytotoxicity and macrophage activation, creating a feedforward immunosuppressive network within the TME. Therapeutic disruption of these pathways (e.g., via Siglec-9 blockade) enhances neutrophil-mediated tumor killing in preclinical models, highlighting this axis as a promising immunomodulatory target.

#### 4.2.3. Dendritic Cells (DCs)

As professional antigen-presenting cells, DCs play a pivotal role in initiating tumor-specific T cell responses and represent crucial targets for immunotherapy [111,112]. Emerging evidence demonstrates that Siglec receptors significantly modulate DC function through multiple mechanisms: (1) Siglec-G impairs cross-presentation capacity in murine DCs by disrupting MHC class I-peptide complex formation [96]; (2) Siglec-E engagement by sialoglycans regulates DC activation thresholds [97]; and (3) tumor-associated DCs from cancer patients exhibit elevated expression of inhibitory Siglecs (-7, -9, and -10) that contribute to immune suppression [95]. Therapeutic targeting of Siglec–DC interactions shows promising immunomodulatory effects: Siglec-7 blockade enhances T cell priming and DC activation in vitro by disrupting glycan-mediated immunosuppressive signaling [113], while the tyrosine kinase inhibitor dasatinib promotes DC migration by reducing Siglec-9/-3 phosphorylation, potentially augmenting chemoimmunotherapy efficacy [114]. These findings highlight the critical balance between Siglec-mediated inhibition and DC activation, suggesting that selective disruption of specific Siglec pathways (particularly Siglec-7/-9) could enhance DC-based antitumor immunity. Further elucidation of Siglec–DC interactions across different cancer types may yield novel combinatorial immunotherapy approaches tailored to overcome microenvironmental immunosuppression.

#### 4.2.4. Natural Killer Cells (NK Cells)

NK cells are specialized lymphocytes that belong to the innate immune system. They do not rely on specific antigen presentation but are able to directly recognize and kill infected cells or tumor cells. NK cells kill target cells directly by releasing cytotoxic molecules. In addition, they are able to secrete various cytokines, such as interferon-gamma (IFN-γ), to regulate the immune response. NK cells recognize target cells by activating and inhibiting receptors on their surface. Activating receptors recognize and bind to stress or mutant molecules on the surface of target cells, while inhibiting receptors bind to MHC-I molecules on the surface of normal cells, preventing NK cells from attacking normal cells. Human NK cells mainly express Siglec-7 and -9 [86], which engage with tumor-associated sialoglycans to suppress NK cell function through multiple mechanisms: (1) Siglec-9 binding to MUC16 on ovarian cancer cells inhibits anti-tumor responses [115]; (2) immune synapse formation induces Siglec-7 ligand accumulation and sustained inhibitory signaling through delayed glycoconjugate endocytosis [100]; and (3) in multiple myeloma, Siglec-7 engagement facilitates tumor escape from NK cell surveillance [9]. These interactions demonstrate how tumor cells exploit the Siglec checkpoint axis to evade NK-mediated immunity. Therapeutic strategies targeting Siglec-7/9 (e.g., antibody blockade or glycan remodeling) may therefore enhance NK cell anti-tumor activity, particularly in Siglec ligand-high malignancies like ovarian cancer and multiple myeloma. The differential expression of Siglec receptors across NK cell subsets (e.g., CD56bright vs. CD56dim) further suggests opportunities for precision immunotherapies tailored to specific tumor microenvironments.

#### 4.2.5. Lymphocyte

The immunosuppressive tumor microenvironment extends beyond myeloid cells to include lymphocytes, with both B and T cells exhibiting Siglec-mediated regulation of anti-tumor immunity. B lymphocytes predominantly express CD22 (Siglec-2), a conserved receptor that modulates B cell activation thresholds through ST6GAL1-catalyzed sialylation, maintaining immune tolerance [16,116]. Clinically, the anti-CD22 monoclonal antibody SM03 has shown efficacy in Phase III trials by disrupting CD22-SHP1-mediated NF-κB suppression, restoring B cell responsiveness in malignancies [117], while CD22-targeted CAR-T therapy demonstrates potent activity against B-cell acute lymphoblastic leukemia [118]. T lymphocytes primarily express Siglec-7 and -9, which engage tumor-associated sialoglycans to inhibit activation and proliferation—particularly in glioblastoma, where Siglec-9 functions as a macrophage-associated immune checkpoint that limits T cell responses to immunotherapy [119].

The broader Siglec–ligand network facilitates immune evasion through multiple tumor-specific mechanisms: (1) CD24-Siglec-10 interactions inhibit phagocytosis in ovarian/breast cancers [120]; (2) SELPLG engages Siglec-7 to promote myeloma immune escape [9]; (3) MUC1/MUC16 O-glycans bind Siglec-9 on monocytes/macrophages in breast cancer [115,121]; and (4) GD3 ganglioside suppresses NK cytotoxicity via Siglec-7 in melanoma [122]. These findings position Siglecs as multifunctional immune checkpoints that tumors exploit through varied glycoconjugate interactions. Therapeutic targeting of specific Siglec–ligand axes (e.g., CD24-Siglec-10 or GD3-Siglec-7) may overcome microenvironmental immunosuppression, with emerging strategies including Siglec-blocking antibodies, glycan remodeling enzymes, and combination approaches with existing immunotherapies. The cell-type-specific expression patterns of Siglecs across lymphocyte subsets further enable precision targeting to restore anti-tumor immunity while minimizing systemic autoimmunity risks.

### 4.3. Feasibility of Early Malignant Tumor Detection Using Sialic Acid and Siglecs

The sialic acid–Siglec axis represents a promising frontier for both early cancer detection and immunotherapy, particularly for tumors resistant to conventional immune checkpoint blockade. As innate immune checkpoints, Siglec receptors and their sialoglycan ligands play critical roles in tumor immune evasion by regulating phagocytosis and immune surveillance [123]. While current immunotherapies targeting PD-1/PD-L1 and CTLA-4 have shown remarkable success in certain cancers [124,125], their efficacy remains limited by immunosuppressive tumor microenvironments and the “cold tumor” phenotype characterized by poor T cell infiltration [126,127]. This therapeutic gap has spurred interest in Siglec-targeted approaches, leveraging their unique expression patterns and mechanisms of immune regulation. Clinically, Siglec-2 (CD22) has emerged as a validated target in B-cell malignancies, with antibody-drug conjugates and bispecific CD19/Siglec-2 CAR-T therapies demonstrating efficacy in relapsed B-cell lymphomas and acute lymphoblastic leukemia [128]. Meanwhile, Siglec-15 has garnered attention as a potential alternative to PD-L1 blockade, with the NC318 antibody currently in Phase I/II trials for PD-1/PD-L1-resistant cancers [129,130]. Beyond direct tumor targeting, Siglec blockade (e.g., anti-Siglec-7/-9/-10) can restore NK/T cell cytotoxicity [109,113,120], while interventions against tumor-associated sialoglycans (e.g., CD24-Siglec-10, GD3-Siglec-7) may enhance phagocytosis and NK activity [120,122]. For early detection, aberrant sialylation patterns and Siglec overexpression in premalignant lesions offer potential as diagnostic biomarkers through liquid biopsies or molecular imaging. However, challenges remain, including tumor heterogeneity in Siglec expression, risks of autoimmune toxicity from systemic blockade, and the need for rational combination strategies with existing therapies [131]. The dual utility of the sialic acid–Siglec axis, as both a detection marker and therapeutic target, positions it as a transformative platform in oncology, particularly for immunologically “cold” tumors refractory to current immunotherapies. Ongoing clinical evaluation of Siglec-directed agents and deeper mechanistic understanding of Siglec biology in the tumor microenvironment will be crucial for realizing this potential and expanding treatment options for cancer patients.

## 5. The Impact of Sialylation on Cancer Development

Hypersialylation, a hallmark of cancer, drives tumor aggressiveness by promoting immune evasion, altering cell adhesion, enhancing metastasis, and conferring resistance to chemo/radiotherapy [132]. This aberrant glycosylation modulates multiple oncogenic pathways across various cancers (pancreatic, colon, breast, ovarian, melanoma, and lung): (1) excessive sialylation of death receptors (FasR, TNFR1) inhibits apoptosis [133,134,135]; (2) sialylated growth factor receptors (e.g., FGFR1) aberrantly activate ERK/FAK signaling, fueling proliferation, angiogenesis, and invasion [133]; and (3) tumor-associated sialylselectin ligands (SLe^a^, SLe^x^, CD44) facilitate hematogenous metastasis by mediating circulating tumor cell adhesion to vascular endothelium [136], which is a mechanism exemplified by SLe^x^-high breast cancers exhibiting preferential metastatic tropism [137].

Clinically, sialic acid-rich glycoconjugates serve as established biomarkers (CA19-9/SLe^x^ in pancreatic cancer; CA125/MUC16 in ovarian cancer; CA15-3/MUC1 in breast cancer) [138], while specific sialyltransferases show prognostic potential. ST6GAL1 correlates with advanced stage, chemoresistance, and metastatic recurrence [139], with therapeutic targeting of ST6Gal-1-high triple-negative breast cancer stem cells demonstrating improved efficacy through glycan engineering approaches [140]. Similarly, ST8SiaII marks metastatic neuroblastoma [141], and GM3 synthase (ST3GAL3) depletion suppresses castration-resistant prostate cancer by reducing cancer stemness and EMT markers [142]. These findings position sialylation enzymes and their products as both diagnostic indicators and therapeutic vulnerabilities across malignancies, with emerging strategies focusing on sialyltransferase inhibition, glycan remodeling, and sialoglycan-targeted immunotherapy to disrupt pro-tumorigenic sialic acid signaling.

## 6. Methods of Targeting Siglec–Sialylation Therapy

The Siglec–sialic acid interaction represents a promising immunotherapeutic target, with multiple intervention strategies under investigation to disrupt this immunosuppressive axis and enhance anti-tumor immunity.

### 6.1. Antibody Therapy

**Figure 5:**
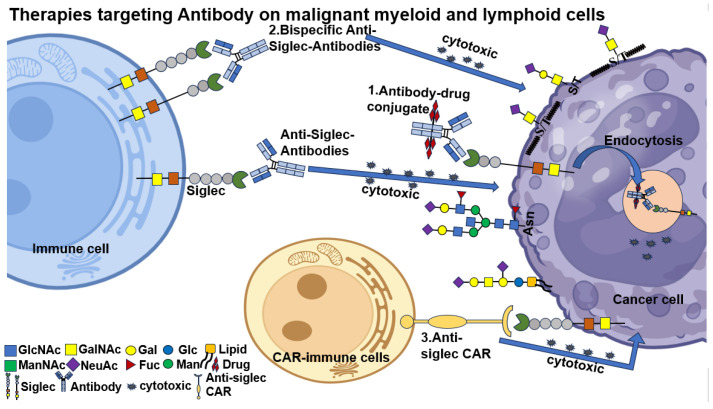
Therapies targeting antibodies on malignant myeloid and lymphoid cells. Targeting antibody therapies include monoclonal antibodies, bispecific antibodies, chimeric antigen T cell (CAR-T) therapy and antibody-drug conjugate. CAR T-cell therapies have been developed that target Siglecs displayed on the surface of cancer cells, leading to cytotoxicity in those cells. Cytotoxic granules are depicted as black dots. Illustrated ADCs are composed of an anti-Siglec conjugated to a cytotoxic small-molecule payload. The antibody portion of the drug targets Siglecs, which are displayed on the surface of cancer cells, leading to internalization of the antibody and the drug and subsequent release of the cytotoxic payload within the cancer cell.

As Siglecs are surface receptors expressed on most immune cells, antibody-based therapies represent a strategic approach to exert cytotoxic effects by targeting immune cells within the tumor microenvironment. These include antibody-drug conjugates (ADCs), anti-Siglec bispecific T-cell engagers (BiTEs), and chimeric antigen receptor (CAR) T-cell therapies (Figure 5).

#### 6.1.1. Monoclonal Antibody (mAb)

Immune checkpoint inhibitors (ICIs) utilizing anti-Siglec mAbs demonstrate dual mechanisms of action: target specificity and antibody-dependent cellular cytotoxicity. These mAbs specifically bind Siglecs on immune cells, thereby enhancing anti-tumor immune responses and attenuating immunosuppression within the TME. Furthermore, they disrupt interactions between tumor-associated sialoglycans and Siglecs, preventing immune evasion. Preclinical studies validate the therapeutic potential of Siglec-targeting mAbs. For instance, Siglec-15, highly expressed on TAMs, represents a promising ICI target due to its role in cancer immune evasion. The anti-Siglec-15 mAb NC318 has demonstrated therapeutic efficacy in preclinical models and is currently undergoing phase I/II clinical evaluation [129]. Additionally, the anti-CD22 mAb SM03 mitigates CD22/SHP1-mediated suppression of NF-κB signaling, restoring B-cell responsiveness in malignancies [117].

Antibody-drug conjugates (ADCs) constitute an emerging class of potent therapeutics comprising mAbs linked to cytotoxic payloads. These agents selectively deliver potent cytotoxins to tumor cells while sparing normal tissues, thereby overcoming key limitations of conventional chemotherapy and providing an improved therapeutic index [143]. ADCs exhibit significant activity against refractory cancers, with several agents receiving clinical approval [144]. FDA-approved ADCs for solid tumors include those targeting HER2, TROP-2, and Nectin-4 [145,146,147]. Siglecs undergo ligand- or antibody-induced endocytosis followed by recycling to the cell surface, rendering them optimal ADC targets [148,149,150]. Several Siglec-targeting ADCs are in development, with some already implemented clinically. For example, gemtuzumab ozogamicin (anti-CD33 ADC) is used for CD33^+^ acute myeloid leukemia, while inotuzumab ozogamicin (anti-CD22 ADC) is for relapsed acute lymphoblastic leukemia [151].

#### 6.1.2. Bispecific Antibody (BsAbs)

BsAbs can recognize and bind to two different antigens or epitopes, redirect immune cells to tumor cells, deliver drugs to tumors, and block two biological pathways important to tumors for cancer treatment [152,153,154]. Fc domain-containing BsAbs engage Fcγ receptors on immune effector cells (e.g., NK cells, monocytes, macrophages), inducing antibody-dependent cellular cytotoxicity. However, complement binding may trigger complement-dependent cytotoxicity, potentially causing nonspecific immune activation during treatment [155]. Among T-cell-engaging BsAbs, bispecific T-cell engagers (BiTEs) represent a clinically validated subclass. BiTEs comprise two single-chain variable fragments (scFvs) from anti-tumor-associated antigen and anti-CD3 mAbs, connected via short linkers [156]. These scFvs—containing immunoglobulin heavy and light chain variable domains—exhibit 100- to 10,000-fold greater tumor cell killing potency than conventional BsAbs or IgG mAbs [157]. AMG330, a CD33/CD3-targeting BiTE, demonstrates significant tumor growth inhibition in acute myeloid leukemia xenograft models [158]. Similarly, CD19/CD22 [159] and CD20/CD22 [160] BsAbs show enhanced efficacy over monospecific targeting. Additionally, Siglec6-targeted T-cell-recruiting BsAbs effectively eliminate chronic lymphocytic leukemia cells while sparing Siglec6^−^-healthy B cells [123].

#### 6.1.3. Chimeric Antigen Receptor T-Cell (CAR-T) Therapeutic Approaches

CAR-T therapy involves genetic modification of patient-derived T cells to express synthetic receptors that recognize specific tumor antigens [161]. These CARs enable precise targeting of cancer cells, enhancing T cell-mediated tumor killing. Following ex vivo expansion, CAR-T cells are reinfused into patients, minimizing host immune rejection. Despite challenges including cytokine release syndrome and limited solid tumor efficacy, CAR-T therapy has achieved remarkable success in hematologic malignancies such as acute lymphoblastic leukemia (ALL) and specific lymphomas [162]. CAR-T cells persist as memory phenotypes, enabling rapid and durable responses superior to conventional therapies [163,164]. FDA-approved CAR-T products target refractory diffuse large B-cell lymphoma and ALL [165].

CAR-T cells targeting Siglec-2 (CD22) and Siglec-3 (CD33) receptors show clinical efficacy against leukemia and lymphoma, respectively [166,167,168]. CD22-directed CAR-T therapy effectively inhibits ALL progression [118]. Recent studies indicate Siglec-6-targeted CAR-T cells induce remission in acute myeloid leukemia (AML) without requiring allogeneic hematopoietic stem cell transplantation [169]. Bispecific CD19/CD22 CAR-T therapy overcomes antigen escape in relapsed/refractory B-ALL, demonstrating durable responses in Phase I trials [170,171]. Additionally, CD33-targeted CAR-NK cells reduce leukemia burden and prevent bone marrow engraftment in AML xenografts, suggesting a novel AML treatment paradigm [172]. A recent Phase I dose-escalation study confirmed durable clinical activity of CD22 CAR-T (CAR22) in patients progressing after CD19 CAR-T therapy, validating CD22 as a salvage target for CAR-T-resistant lymphomas [173]. These advances underscore the significant clinical potential of CAR-T approaches.

### 6.2. Siglec Inhibitor

In recent years, significant efforts have been directed toward developing Siglec blockers as cancer immunotherapies. These agents function by disrupting inhibitory interactions between immune cell Siglecs and their ligands expressed on cancer cells, thereby blocking the immunosuppressive effects of inhibitory Siglecs while simultaneously promoting immune activation. Recent studies demonstrate that inhibiting GD2 binding to macrophage Siglec-7 enhances anti-tumor immunity, further increasing phagocytosis and amplifying the efficacy of CD47 blockade [174].

### 6.3. Sialylation Inhibitor

**Figure 6:**
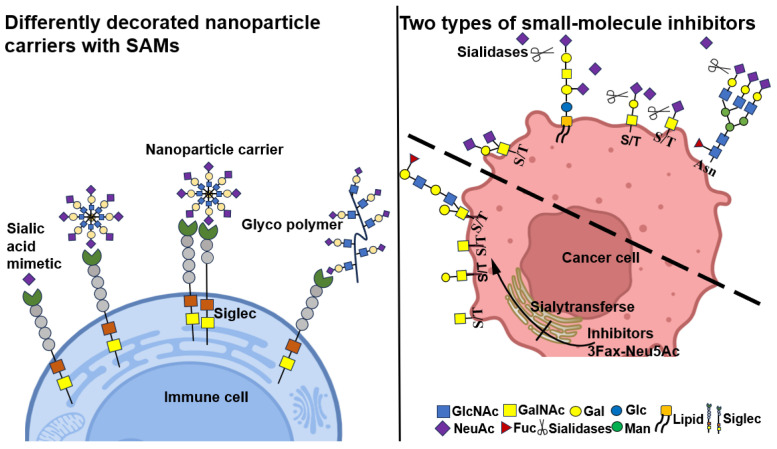
Differently decorated nanoparticle carriers with SAMs and two types of small-molecule inhibitors. Carbon groups (C-) in the sialic acid backbone that could be substituted to design high-affinity SAMs. The nanoparticle carriers decorated with sialic acid mimetics for high-affinity binding to Siglecs and Membrane-targeting salivary glycopolymers can bind to Siglec on immune cells with high specificity. Immunosuppression of siglec–sialic acid interactions is prevented by the application of small-molecule inhibitors targeting sialidase and sialyltransferase, which reduce or hinder sialic acid synthesis and prevent siglec–sialic acid interactions.

Altering the synthesis and expression of sialoglycan groups while reducing sialoglycan density in tumor cells and the tumor microenvironment represents an alternative approach to block immunosuppressive effects. This includes utilizing sialic acid mimics to inhibit sialic acid biosynthesis and developing sialic acid biosynthesis inhibitors. Sialidase treatment may also influence cancer progression by modifying signaling in tumor cells and immune cells (Figure 6).

#### 6.3.1. Sialtransferase Inhibitors

Enzymatic reduction in sialoglycan density in tumors has been tested and is currently under investigation in human clinical trials. An αHER2 antibody–sialidase conjugate efficiently and selectively removes diverse sialoglycans from breast cancer cells. In syngeneic murine breast cancer models, desialylation enhanced immune cell infiltration and activation while extending survival time [175]. This intervention repolarized TAMs, bolstering anti-tumor immunity and inhibiting tumor progression. Another study chemically fused recombinant sialidase with trastuzumab, a human epidermal growth factor receptor 2 (HER2)-specific antibody. This antibody–sialidase conjugate mediated HER2-dependent desialylation, reducing natural killer (NK) cell binding to inhibitory Siglec receptors and enhancing antibody-dependent cellular cytotoxicity (ADCC) in tumor cells [176].

#### 6.3.2. Sialic Acid Mimics (SAMs)

SAMs are synthetic molecules structurally mimicking sialoglycoproteins that function as high-affinity Siglec ligands. SAMs can either block tumor cell-expressed Siglecs or competitively inhibit natural Siglec binding, thereby disrupting Siglec–ligand interactions to modulate Siglec signaling and immune responses in disease contexts [177]. Another promising strategy involves intratumoral injection of SAMs to locally block sialic acid expression. This approach increased tumor-infiltrating NK cells and CD8^+^ T cells while reducing regulatory T cells and myeloid-derived suppressor cells, ultimately enhancing CD8^+^ T cell-mediated cytotoxicity and suppressing tumor growth [178].

The fluorinated sialic acid analog Ac_5_3FaxNeu5Ac disrupts sialic acid expression in tumor cells [179]. By inhibiting sialyltransferases (STs), impairing sialic acid–glycan binding, and modulating cellular sialic acid content, studies demonstrate that Ac_5_3FaxNeu5Ac-mediated sialic acid blockade in dendritic cells enhances CD8^+^ T cell proliferation induced by mouse bone marrow-derived DCs [180].

Additionally, SAMs show potential in drug delivery applications. Nanoparticles incorporating CD22-targeting SAMs have been designed to deliver cytotoxic agents (e.g., doxorubicin) to CD22-expressing malignant B cells [181]. Sialic acid-conjugated liposomes enable neutrophil/monocyte-mediated tumor homing of epirubicin for targeted therapy [182]. Furthermore, Bull et al. demonstrated that encapsulation in tumor-targeting nanoparticles prevents metastasis in murine lung cancer models while circumventing systemic toxicity associated with global sialylation inhibition [183].

#### 6.3.3. Development of Novel Sialylation Materials

High-affinity binding is essential for receptor aggregation and subsequent Siglec signaling when competing with natural ligands. A promising alternative involves polyvalent displays of SAMs achieved by either conjugating SAMs to nanoparticles or polymers or modifying living cell glycocalyx via bioorthogonal synthesis [132,184]. SAM-modified nanoparticles, including liposomes, gold nanoparticles, and dendrimer/PLGA-based systems, have been utilized for targeted delivery to Siglec-expressing cells [185] or to modulate Siglec signaling through glycan-decorated surfaces [186,187]. Cells bearing these mucin-mimetic glycopolymers exhibit significantly enhanced Siglec-7 binding and protection from Siglec-7^+^ NK cell-mediated killing. Subsequent investigations revealed concurrent phosphorylation of Siglec-7, SHP-1 recruitment, and suppression of NK cell cytotoxic activity. These findings suggest that soluble or membrane-incorporated glycopolymers may function as SAM carriers capable of engaging immune cell Siglecs and triggering immunosuppressive signaling. Further studies are warranted to validate their biological potential in in vivo cancer models.

## 7. Conclusions and Perspectives

This review summarizes specific alterations in glycosylation, particularly sialylation in cancer. Sialic acid is ubiquitously expressed across all cell types, with abnormally elevated levels serving as a hallmark of malignant transformation. Aberrant sialylation disrupts cell–cell interactions and signaling pathways, driving immune dysfunction that impairs natural killer cell and macrophage activity. These alterations facilitate immune evasion and promote chronic inflammation.

Recent research underscores the critical regulatory role of Siglec receptors in immune cell function. The Siglec–sialoglycan axis represents a promising immune checkpoint target for cancer immunotherapy, modulable through either Siglec receptor blockade or sialoglycan cleavage via sialidases.

Targeting sialoglycans and immune cell Siglec receptors has emerged as a novel strategy for cancer diagnosis and treatment. Antibody-based and glycan-directed approaches have established Siglecs as attractive therapeutic targets for hematological malignancies including lymphoma and leukemia. We further synthesize recent advances in sialic acid inhibitors, providing systematic guidance for future clinical translation and development of next-generation therapeutics. Several inhibitors targeting specific Siglecs (notably Siglec-15) are currently undergoing clinical evaluation. However, comprehensive understanding of Siglec inhibitors’ efficacy profiles, potential side effects, and combinatorial potential with standard therapies remains to be fully elucidated.

As research progresses, sialic acid-targeted therapies hold significant promise for transforming cancer treatment. Therefore, rigorous investigation of sialylation mechanisms using advanced methodologies and contemporary detection technologies is increasingly imperative.

## Figures and Tables

**Table 2:** Preclinical evidence of Siglec-mediated immune modulation in the tumor microenvironment.

Source of Immune Cell	Immune Cells Involved	Siglecs Involved	Effect on Tumor Progression/Antitumor Immunity	Key References
myeloid cell	Tumor-associated Macrophages (TAMs)	Siglec-E, Siglec-1, Siglec-10, Siglec-9, Siglec-11, Siglec-3, Siglec-16, Siglec-15	Polarization into pro-tumorigenic M2- like macrophages, inhibition of phagocytosis (‘don’t eat me signal’)	[92,93,94,95]
myeloid cell	Tumor-associated Neutrophils (TANs)	Siglec-9 (Siglec-E)	Inhibition of ROS production	[92]
myeloid cell	Dendritic cells	Siglec-E, Siglec-G, Siglec-7, Siglec-9, Siglec-10, Siglec-15	Inhibition of antigen presentation	[96,97,98]
Lymphocyte cell	Tumor-infiltrating T cells	Siglec-7, Siglec-9, Siglec-5, Siglec-14	Inhibition of T cell activation and	[99]
Lymphocyte cell	NK cells	Siglec-7, Siglec-9	Inhibition of NK cell-mediated tumor cell killing	[9,100,101,102]

## Data Availability

Not applicable.
